# A case report of pancreatic lymphoepithelial cyst

**DOI:** 10.1097/MD.0000000000045183

**Published:** 2025-10-17

**Authors:** Jingjing Gong, Jiameng Liu, Chi Xu, Yiqiu Shi, Yuwen Shen, Kefu Liu

**Affiliations:** a Department of Medical Imaging, The Affiliated Suzhou Hospital of Nanjing Medical University, Gusu School of Nanjing Medical University, Suzhou, Jiangsu Province, China; b Department of Pathology, The Affiliated Suzhou Hospital of Nanjing Medical University, Gusu School of Nanjing Medical University, Suzhou, Jiangsu Province, China; c Department of Medical Imaging, Suqian First People’s Hospital Affiliated to Nanjing Medical University, Suqian, Jiangsu Province, China.

**Keywords:** computed tomography, pancreatic cystic tumor, pancreatic lymphoepithelial cyst, rare disease

## Abstract

**Rationale:**

Pancreatic lymphoepithelial cyst (PLEC) is a rare benign pancreatic cystic lesion that poses considerable diagnostic challenges owing to its overlapping clinical and imaging features with malignant neoplasms. Misdiagnosis frequently results in unnecessary surgical interventions. This case report aims to elucidate specific clinical and radiological characteristics essential for the accurate diagnosis of PLEC.

**Patient concerns:**

A male patient presented with abdominal pain. Laboratory tests revealed elevated serum carbohydrate antigen 19-9 (CA19-9) levels. An abdominal computed tomography (CT) scan identified a cystic mass in the head and body of the pancreas.

**Diagnoses:**

The patient underwent a partial pancreatectomy and splenectomy, followed by histopathological biopsy, which confirmed the diagnosis of PLEC.

**Interventions:**

Following postoperative PLEC diagnosis, the patient was placed under regular surveillance, including abdominal CT scans and serum CA19-9 monitoring.

**Outcomes:**

The patient underwent partial pancreatectomy and splenectomy without complications. Abdominal pain completely resolved by postoperative day 18. By day 30 of follow-up, CA19-9 levels had decreased to 9 U/L. Subsequent evaluations at 6 months, including abdominal CT and tumor marker assessments, confirmed sustained remission without evidence of recurrence, indicating an excellent clinical outcome.

**Lessons:**

PLEC, a rare benign lesion, often mimics malignancy, potentially leading to unnecessary radical surgeries. Diagnostic hallmarks include its tendency to present as an exophytic cystic mass in middle-aged men; CT features demonstrating cyst fluid density exceeding that of simple or mucinous cysts with thin, mildly enhancing walls; and pathognomonic magnetic resonance imaging findings of T2-hyperintense subcapsular nodules exhibiting restricted diffusion in a noninfiltrative, wall-apposed pattern without ductal communication. Markedly elevated CA19-9 levels should not preclude PLEC. When this constellation of features is present, PLEC should be considered in the differential diagnosis.

## 1. Introduction

Pancreatic lymphoepithelial cyst (PLEC) is an exceptionally rare benign tumor accounting for approximately 0.5% of pancreatic cystic lesions. It was first described as a histopathological entity by Lüchtrath and Schriegers in 1985 as characterized by keratinizing squamous epithelium enveloped by lymphoid tissue.^[1–3]^ Its pathogenesis remains poorly understood; however, it is considered to arise primarily from squamous metaplasia of ductal epithelium, ectopic pancreatic tissue, or the embryonic fusion of branchial cleft remnants.^[4]^ Owing to its low incidence and the clinical and radiographic features that resemble those of malignant tumors,^[5]^ distinguishing PLEC from pancreatic malignancies can be exceedingly challenging, often complicating the selection of appropriate treatment and leading to unnecessary surgeries. Despite documentation in various case reports,^[5–11]^ standardized diagnostic criteria have yet to be established. Current literature identifies recurrent characteristics, including a predilection for middle-aged to older men, well-circumscribed exophytic cystic-solid morphology, thin walls with mild contrast enhancement, infrequent calcification, and cyst fluid attenuation exceeding that of simple fluid density. However, significant ambiguities persist regarding potentially misleading features, such as the presence of mural nodules, diffusion restriction patterns in these nodules, and elevated serum carbohydrate antigen 19-9 (CA19-9) levels, all of which potentially contribute to misdiagnosis as malignancy.^[8]^ This report details the clinical, imaging, and pathological features of a 68-year-old man with PLEC.

## 2. Case presentation

A 68-year-old man presented with a 4-hour history of epigastric pain. Laboratory tests revealed CA-199 levels (310.40 U/mL, normal < 35.00 U/mL).

### 2.1. Computed tomography (CT)

**Figure 1.**
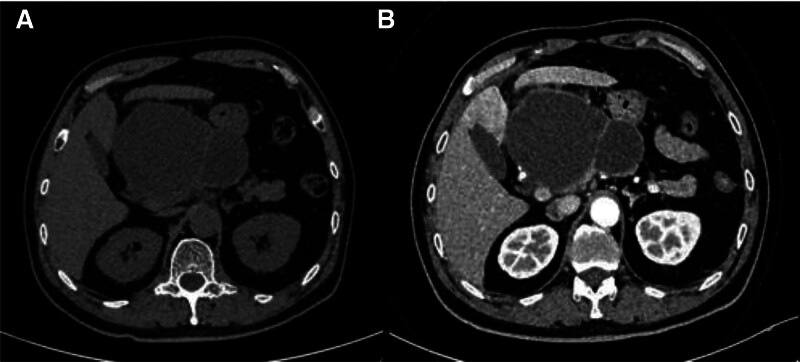
Noncontrast (A) and arterial phase CT (B) scans showing a cystic mass in the head and body of the pancreas. The density of the cystic fluid is slightly higher than that of water, with a few patchy areas of slightly higher density floating within. The cyst wall and septa show mild enhancement, while the contents do not enhance. CT = computed tomography.

A single large cystic lesion was identified in the head and body of the pancreas, characterized by clear boundaries and measuring approximately 125 × 86 × 103 mm (Fig. 1). The average CT value was approximately 35 Hounsfield units. Small patchy areas of slightly higher density and fine septa were observed within the lesion. The wall of the cyst was slightly thickened, demonstrating mild enhancement of the lesion wall and septa upon contrast administration, with no enhancement of the contents.

### 2.2. Magnetic resonance imaging (MRI)

**Figure 2.**
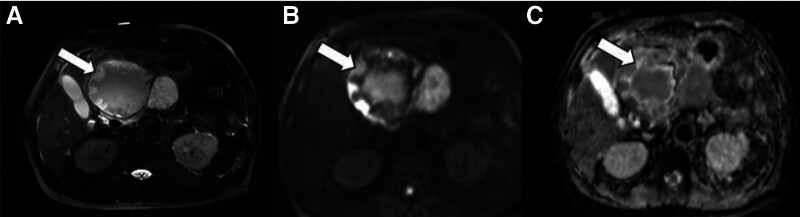
T2-weighted MRI abdomen (A) showing the lesion slightly hyperintense signal, with extensive nodule-like areas of high signal beneath the cyst wall. Diffusion-weighted imaging (B) and apparent diffusion coefficient (C) showing restricted diffusion (white arrow). The cystic fluid also shows restricted diffusion, accompanied by thicker septa without restricted diffusion. MRI = magnetic resonance imaging.

The lesion exhibited a cystic-solid appearance, with the contents displaying an isointense signal on T1-weighted imaging (T1WI) and a slightly hyperintense signal on T2-weighted imaging (T2WI) (Fig. 2). Extensive nodule-like areas beneath the cystic wall exhibited an isointense and hyperintense signals on T1WI and T2WI, respectively. Both the nodule-like areas beneath the cystic wall and the cystic fluid demonstrated restricted diffusion, accompanied by thicker septa lacking restricted diffusion. The lesion did not communicate with the pancreatic duct, and no dilation of the pancreatic or bile ducts was observed, nor were there enlarged lymph nodes in the surrounding area.

### 2.3. Pathology

**Figure 3.**
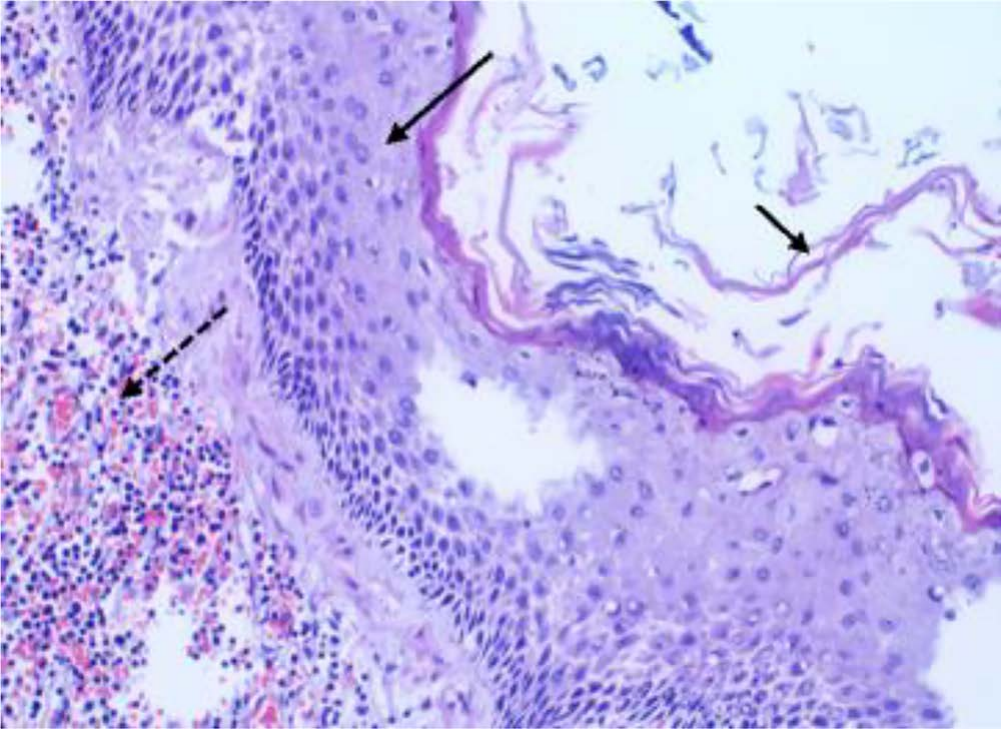
Microscopic examination (HE × 20) reveals the pancreatic cyst lined by stratified squamous epithelium (long black arrow), with strips of keratinized material beneath the cyst wall (short black arrow). Beneath the squamous epithelium, there is lymphoid hyperplasia with formation of lymphoid follicles (long dashed arrow). HE = hematoxylin-eosin staining.

The cyst wall was lined by stratified squamous epithelium (indicated by long black arrows), surrounded by lymphoid aggregates featuring prominent lymphoid follicles and germinal center formation (denoted by long dashed arrows). Linear keratotic material was evident within the cyst lumen (marked by short black arrows). Additionally, vascular proliferation was noted, while no sebaceous glands or cutaneous adnexal structures were identified. The pancreatic resection margin and spleen exhibited no pathological involvement (Fig. 3). The final diagnosis was PLEC.

Post-surgery, the patient’s abdominal pain resolved completely. At the 1-month follow-up, serum CA19-9 levels decreased to 9 U/mL and remained within normal limits. A surveillance abdominal CT scan at 6 months revealed no evidence of recurrence at the surgical site.

## 3. Discussion

PLEC, a rare cystic lesion, potentially originates from squamous metaplasia of ductal epithelium, ectopic pancreatic tissue, or the embryonic fusion of branchial cleft remnants.^[4]^

The pathological features of PLEC dictate its imaging characteristics: lesions can be readily enucleated from the pancreas upon pathological gross examination, presenting as solitary, exophytic, well-circumscribed cystic masses on imaging.^[1]^ This contrasts with cystic changes in pancreatic ductal adenocarcinoma, a malignant tumor typified by ill-defined masses that often involve adjacent organs, and metastasize to lymph nodes.^[12]^

In our case, mild wall thickening and enhancement histologically correlated with proliferative capillaries but pathologically deviated from tumor-induced angiogenesis.^[13]^ This finding differentiates PLEC from simple pancreatic cysts, which exhibit unilocular alterations without walls or septation.^[14,15]^ In addition, PLEC can be distinguished from pseudocysts, which possess uneven walls and are often associated with specific clinical histories, such as acute pancreatitis or abdominal trauma.^[16]^

PLEC is characterized by the presence of cholesterol crystals, keratin debris, and caseous material within the cyst,^[10,13]^ which reflecting in the imaging manifestations, particularly the changes in the noncontrast CT density. Analysis of imaging manifestations in numerous PLEC cases indicates a higher CT attenuation value than that in pancreatic mucinous cystadenoma, serving as a critical discrimination.^[6,9]^ Mucinous cystadenomas are more common in middle-aged women, whereas PLEC is more prevalent in middle-aged men.

Restricted diffusion within T2-hyperintense subcapsular nodules may arise from viscous microenvironments created by densely packed lymphoid follicles and restricted water diffusion owing to keratinaceous debris.^[10]^ Notably, Raval et al reported mucin-secreting epithelium in 78% of PLEC cases, where elevated protein content accounts for the nodular hyperintensity observed on T1WI and T2WI. Crucially, this diffusion restriction is fundamentally distinct from that observed in mucinous cystic adenocarcinoma: PLEC exhibits wall-adherent growth with discrete margins, contrasting with the infiltrative borders typical of malignancies, a pivotal diagnostic distinction.

Although elevated CA19-9 may mimic malignancy, they likely result from cytokine-mediated epithelial secretion and display a positive correlation with lesion size.^[7,10]^ Another discriminator between PLEC and pancreatic malignancies is that CA19-9 levels are significantly lower in the former than in the latter.^[17,18]^

Currently, PLECs are predominantly documented in case reports. Following surgical resection, patients typically experience symptom resolution with no documented recurrences, and tumor markers normalize postoperatively, indicating a favorable prognosis. The literature does not report cases of postoperative recurrence or distant metastases.^[11,19]^ Certain limitations in our study warrant consideration. First, as a single-case-report, the findings may lack generalizability to a broader population. A larger cohort is necessary to substantiate clinical significance. Second, imaging evaluation relied solely on CT and MRI; endoscopic ultrasound and positron emission tomography-computed tomography were not performed, potentially limiting a more comprehensive diagnostic workup. Finally, while follow-up over nearly 2 years indicated favorable outcomes, the absence of long-term monitoring restricts a full assessment of the natural history and recurrence risk of PLEC.

## 4. Conclusion

The characteristic imaging features of PLEC include a large, well-defined, exophytic cystic (solid) lesion in the pancreatic region with a slightly thickened wall. It is not associated with enlarged lymph nodes or dilation of the pancreatic and bile ducts. The cystic fluid demonstrates more pronounced Hounsfield unit attenuation on noncontrast CT scans, while MRI reveals restricted diffusion of the cystic fluid and extensive nodule-like areas beneath the cyst wall accompanied by thicker septa without restricted diffusion. Recognition of these characteristic imaging features is crucial to prevent misdiagnosis as a surgical lesion, thereby avoiding unnecessary pancreatectomy, which carries significant morbidity.

## Author contributions

**Data curation:** Jiameng Liu, Yiqiu Shi.

**Investigation:** Chi Xu, Yuwen Shen.

**Writing – original draft:** Jingjing Gong.

**Writing – review & editing:** Kefu Liu.
